# A Visual Measurement of Water Content of Crude Oil Based on Image Grayscale Accumulated Value Difference

**DOI:** 10.3390/s19132963

**Published:** 2019-07-05

**Authors:** Qing Liu, Bo Chu, Jinye Peng, Sheng Tang

**Affiliations:** School of Information and Technology, Northwest University, Xi’an 710127, China

**Keywords:** crude oil, water content, computer vision, image processing

## Abstract

In the process of oil exploitation, the water level of an oil well can be predicted and the position of reservoir can be estimated by measuring the water content of crude oil, with reference for the automatic production of high efficiency in the oil field. In this paper, a visual measuring method for water content of crude oil is proposed. The oil and water in crude oil samples were separated by centrifugation, distillation or electric dehydration, and a water–oil layered mixture was formed according to the unequal densities. Then the volume ratio of water and oil was analyzed by digital image processing, and the water content and oil content was able to be calculated. A new method for measuring water content of crude oil based on IGAVD (image grayscale accumulated value difference) is proposed, which overcomes the uncertainty caused by environmental illumination and improves the measurement accuracy. In order to verify the effectiveness of the algorithm, a miniaturization and low-cost system prototype was developed. The experimental results show that the average power consumption is about 165 mW and the measuring error is less than 1.0%. At the same time, the real-time and remote transmission about measurement results can be realized.

## 1. Introduction

Crude oil moisture content is a vital parameter in the petroleum exploitation and petrochemical industries, and it has significant reference value to crude oil processing, storage, transportation, and pricing. By measuring the water content of crude oil, the position of reservoir can be estimated in time [1,2,3]. For measurement of water in crude oil, there are two major categories which are online and offline [4].

In 2009, a method for the determination of water content in crude oil mixture by microwave technology was proposed by Makeev et al. [5,6]. Sharma et al. did more research on this method to a high degree [7]. In 1995, García-Golding et Al. measured water content in crude oil using the conductivity principle [8]. Chang et al. studied a method of measuring water content in crude oil based on ultra-short-wave technology in 2008 [9]. In 2008, Zhao et al. proposed spectral absorption method to measure water content of crude oil [10]. A high-resolution capacitive sensing system was proposed by Aslam et al. in order to measure water content of crude oil in 2014 [11,12]. The methods mentioned above are online measuring methods. They can measure water content accurately in crude oil samples in real and short time and reduce the labor intensity in the production process. However, online methods have some insurmountable disadvantages. They have stringent requirements on the measuring environment and devices, such as the alkanes and hydrocarbons in crude oil, and the temperature of the environment also has a negative influence on the measurement results.

The traditional distillation, centrifuge, electric dehydration, as well as the Karl-Fischer titration belong to the offline measurement methods [13,14,15,16,17]. The main principle of distillation is to separate water from petroleum in crude oil samples by a certain chemical substance. On this basis, the volume of water and oil are measured separately to calculate the water content of crude oil. For the Karl-Fischer titration, quantitative chemical reagents are added to the crude oil to measure the water content by chemical reaction [15,16,17]. Offline methods are classical methods. In recent years, those methods have been improved significantly in terms of accuracy and application scope. Those methods are reliable and less sensitive to oil emulsification. However, the measuring process of the offline measurements is complicated and the measuring cycle is longer compared to online measurements. 

In recent years, digital image processing has become a popular and attractive research area. The technology has been widely used in artificial intelligence, industrial detection, and so on, and has promoted the development of relevant disciplines [18,19,20]. Computer vision provides another new way to measure water content of crude oil. Over the years, many research efforts have been performed to estimate the level of liquid in transparent containers. For example, Takagi et al. designed a liquid level measuring device based on computer vision technology [21]. They detected the horizontal position of the liquid level by locating the inflection point on the liquid interface of slanted metal strip placed in the liquid. Batagelj et al. proposed an automatic calibration method based on computer vision technology for liquid level in glass thermometers [22]. Chakravarthy et al. studied an optical imaging system based on computer vision to detect the liquid level in a closed container [23]. Beyond those, a liquid level measuring device on image processing technology was proposed by Yu [24]. In addition, Wang [25] as well as Eppel [26] studied the applications of computer vision in liquid level detection. The horizontal liquid level detection technology on computer vision is increasingly popular and belongs to the typical non-contact liquid level measurement. Compared with the contact liquid level measurements, this method can be effectively applied to the liquid level measurement of corrosive and explosive liquids as well as other special application scenarios.

The methods of measuring water content mentioned above are based on the principle that the physical or chemical properties of each component in crude oil sample are different. They have gained excellent achievements in practical application. Evans et al. indicated that visualization technology is indispensable and crucial for the development of the oil and gas industry [27]. An increasing number of relevant studies are well adapted to the development trend of automation and intellectualization in the field of petroleum. Guilherme et al. applied cutting image analysis to petroleum well drilling monitoring in order to assist the drilling process [28]. Wang et al. studied a visualization method to detect gas–oil–water flow in horizontal pipelines [29]. Sun et al. proposed two geovisualization methods, associating the spatial information of wells with rules in oil and gas well data to gain a better understanding of oil well conditions [30]. 

However, the methods based on image processing are rarely found in existing references for the measuring water content in crude oil. By referring to these already used methods, the method proposed in this paper combines computer vision with water content measuring method and extends computer vision to a new application field. This method can automatically measure after separating water–oil from the sample. It is slightly different from the traditional methods as most of traditional off-line methods measure the content manually. The proposed method is a novel non-contact automatic measurement technology. A prototype was designed in order to verify the feasibility and validity of the method and its technical indicators. The prototype of system principle is currently used in laboratory measurement. In the ensuing research, we will optimize the prototype. This method is suitable for the scenarios where transparent containers are used to hold the samples after separating oil and water. 

It needs to be considered that timely measurement is extremely crucial for predicting the water level in the well and estimating the position of the oil layer. However, the geographical locations of the oil fields are relatively remote and it is difficult to ensure that professional technicians would work all day on the job. Therefore, remote data transmission and device control have been added to the measurement system to further improve the automation of the device after fulfilling accurate measurement for water content of crude oil.

This paper is organized as follows. In Section 2, the proposed method for the water content of crude oil using computer vision is discussed. In Section 3, the implementation of the proposed method is discussed and a prototype of the system is developed. In Section 4, the system performance test is introduced and the test accuracy of the new method proposed in this paper is measured to verify the effectiveness of this method. Finally, some conclusions are drawn in Section 5.

## 2. Measurement for the Water Content of Crude Oil Based on Computer Vision

### 2.1. Method Overview

**Figure 1:**
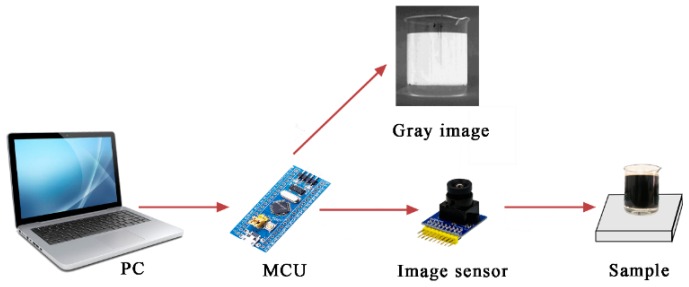
Block diagram of water content of crude oil measurement based on computer vision.

The technical scheme on measuring the water content in crude oil based on computer vision presented in this paper is shown in Figure 1 and can be specifically expressed as follows. First, in order to promote the separation of oil and water molecules, the crude samples were treated by electric dehydration. Then, the oil phase and the water phase were separated, and a water–oil layered mixture having a clear color difference was obtained. On this basis, a low-cost general-purpose image sensor was used to collect the RGB image of the mixture and the transparent container, and the microprocessor converted the RGB image into a grayscale image. The grayscale accumulated value difference (IGAVD) algorithm was used to detect the interfaces of air–oil, oil–water, and water–container bottom, as well as obtain their corresponding coordinate positions. Finally, the water content and oil content in the sample were measured according to two-dimensional image plane correspondence. The IGAVD algorithm improved the applicability of the measuring device to some extent, which overcame the uncertainty caused by environmental factors and improved the measurement accuracy.

### 2.2. Proposed Algorithm

The corresponding coordinate positions of each boundary can be detected by the IGAVD algorithm to calculate the water and oil contents in the crude oil sample. The algorithm includes three steps: Image preprocessing, accumulating the gray values, and finding first derivative of the accumulations.

#### 2.2.1. Image Preprocessing

**Figure 2:**
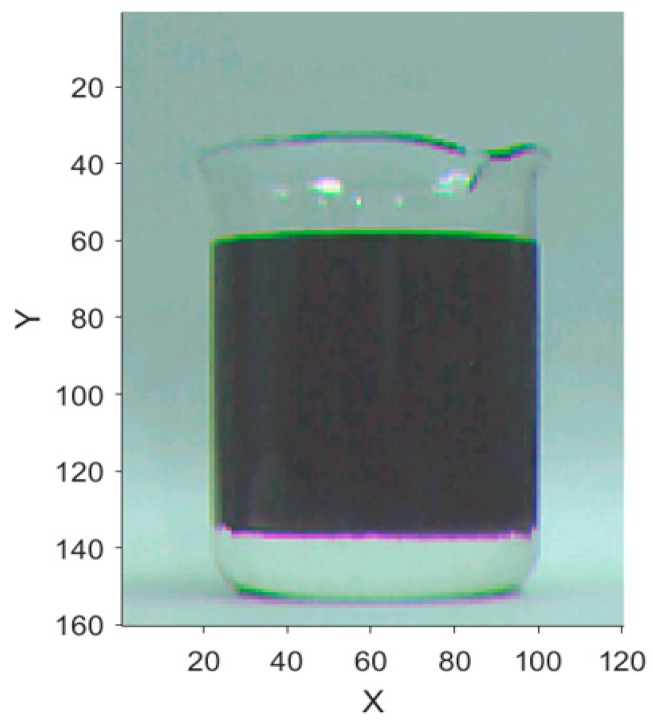
RGB image of the crude oil to be tested.

A low-cost, general-purpose image sensor was used to acquire RGB image of the transparent container containing layered crude oil sample, as shown in Figure 2. A frame of image was stored in FIFO (first in first out). MCU synchronized FIFO timing sequences to read the image data collected by the sensor, and then performed image data processing [31]. Using FIFO to buffer image data can reduce the frequency requirement of the image processing unit, so that general MCU can be used.

The RGB image contains the information of luminance, and chrominance, each color is composed of red, green, and blue superimposed in unequal intensities [32,33,34]. Each pixel can be regarded as a three-dimensional vector. RGB image has some disadvantages, such as cumbersome processing, long operation cycle, and large storage memory. In practical applications, the spatial uniformity of RGB images is inferior, and changes in color are difficult to detect by image sensors [20,35,36,37]. In the actual measurement, it was found that the image in RGB was affected by environmental factors greatly, and the edges could not be identified accurately and quickly. It was obvious that encoding image data in RGB format could not fulfill the need of efficient and reliable requirements in practical application of oil fields.

YUV is a color space commonly used in computer vision. Some studies proved that YUV color space is more suitable for computer vision than RGB color space [34,38]. Y channel in YUV color space represents luminance, U and V represent chrominance and chroma and carry color information. YUV color space separates luminance and chrominance [39,40,41]. For a human visual system, luminance is often more important than chrominance and chroma, and so is computer vision. In RGB color space, luminance, chrominance, and chroma are combined together, and some small changes are difficult to be recognized by the computer. In order to have a better application about computer vision, based on the variation relationship between RGB and YUV color space, the correspondence between Y, U, V, and R, G, B color components of pixels is established [40,42].

(1) $$\left[\begin{array}{l} Y\\ U\\ V \end{array}\right]=\left[\begin{array}{ccc} 0.299 & 0.587 & 0.114\\ -0.196 & -0.332 & 0.500\\ 0.500 & -0.419 & -0.813 \end{array}\right]\left[\begin{array}{l} R\\ G\\ B \end{array}\right]$$

Li et al. [43] indicated that Y component in YUV color space can describe the grayscale value of the image. The grayscale value is a constrained linear combination of R, G, and B channels of the input color image. The weights of R, G, and B are 0.299, 0.587, and 0.114, respectively, and their summation is 1.

(2) $$Gray_{0}=R\times 0.299+G\times 0.587+B\times 0.114$$

Grayscale image contains no chrominance and chroma information, only luminance, each pixel is equivalent to a one-dimensional vector [20,44]. From the perspective of the overall brightness distribution feature of the image, the grayscale image and the RGB image describes the consistent feature. Compared with RGB image, the amounts of redundant information and image data to be processed in grayscale image are reduced, and the storage memory is lower, so that the general-purpose MCU can quickly and accurately process the collected water–oil layered mixture image.

MCU Arithmetic Logic Unit calculating speed about floating-point is slow. Equation (3) enlarges the floating-point coefficients of Equation (2) by 1000 times and converts them into integer, and then reduces the coefficients by 1000 times to ensure the same conversion relationship between RGB and gray images. For Equation (3) to be rounded, 500 is added to the divisor.

(3) $$Gray_{1}=\frac{R\times 299+G\times 587+B\times 114+500}{1000}$$

However, processing data with Equation (3) could cause some problems, such as slow data calculating speed and low accuracy, which could not meet the basic requirements of water content testing. The MCU Arithmetic Logic Unit has a better mastery of handling integer and shift operations than division. As shown in Equation (4), firstly, the coefficients 0.299, 0.587, and 0.114 of the three components R, G, and B in Equation (2) are expanded by 65,536 (2^16^) times and then rightly shifted by 16 bits to achieve the reduction of the corresponding coefficient, which is substantially divided by 65,536 (2^16^). The symbol ≫ denotes right-shift operation.

(4) $$Gray_{2}=(R\times 19,595+G\times 38,469+B\times 7472)\gg 16$$

In relation with the features of Arithmetic Logic Unit, Equation (4) combines integer operation with shift operation, and it is used in MCU program running on the prototype. The operations in Equation (4) could shorten the conversion time from RGB to gray, save the memory of MCU, and reduce the energy consumption of the device. These advantages could accord with the requirements of fast speed, high precision, and low energy consumption in measuring process to a certain extent.

**Figure 3:**
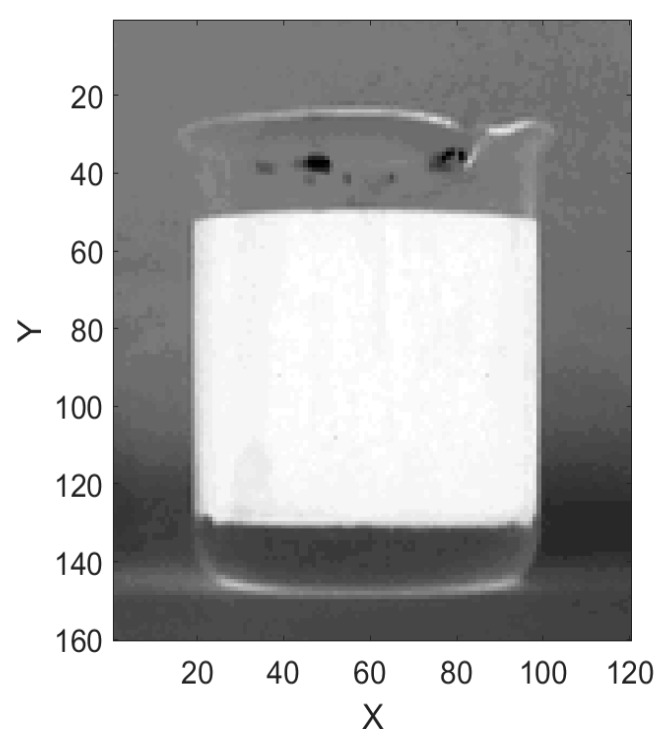
Grayscale image of crude oil to be tested.

The gray image is shown in Figure 3. In the RGB image, the upper oil part of the water–oil layered mixture is darker and the lower water part is lighter in color. In Figure 3, it can be seen that the oil is brighter and it is distinguished more easily. For a 256-level grayscale image, the white grayscale value is the largest, which is 255, and the black grayscale value is the smallest, which is 0 [20]. Converting RGB image to gray image makes MCU easier to process the data. 

#### 2.2.2. Accumulating the Gray Values 

As can be seen in Figure 3, the gray image shows that the oil–water interface is still fuzzy. This is due to the influence of electronic and environmental noise.

IGAVD algorithm accumulates gray value by Equation (5) for the purpose of reducing the impact on image.

(5) $$C(y)=\sum_{x=0}^{m-1}F(x,y)$$

*C(y)* denotes the total summation of the accumulated gray value of the horizontal pixels, *F(x, y)* represents the grayscale value of each pixel in the two-dimensional image, and *m* is the total number of the horizontal pixels.

**Figure 4:**
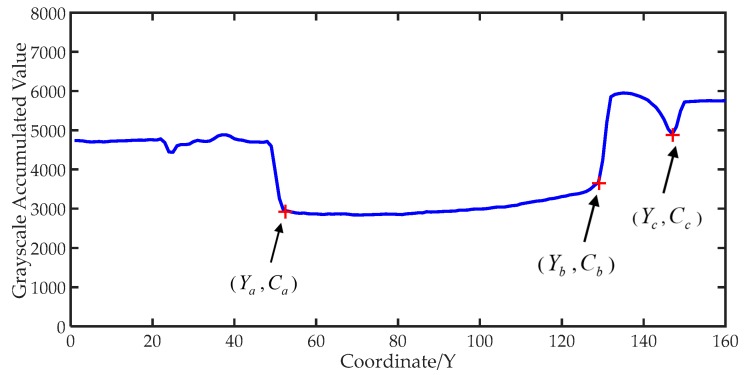
The graph of image accumulated grayscale value.

The algorithm accumulates the grayscale of each horizontal pixel according to the same Y-axis in Figure 3, the data curve of accumulated value is shown in Figure 4. Finally, each Y-axis corresponding grayscale accumulated value is stored in RAM of the MCU. The influence of noise is weakened to some extent by accumulating the grayscale value, the edges of the image are enhanced in this way as well. 

Where ***Y_a_*** is the coordinate position of the air–oil boundary, ***Y_b_*** is the coordinate position of the oil–water boundary, ***Y_c_*** is the coordinate position of the container’s bottom. ***C_a_***, ***C_b_***, and ***C_c_*** are the grayscale accumulated values of the corresponding positions.

#### 2.2.3. Finding First Derivative of the Accumulations

It can be seen in Figure 4 that the grayscale accumulated value of the upper oil part is lower and the grayscale accumulated value of the water part is higher. The corresponding coordinates of the concave portion in Figure 4 are the positions of oil in the two-dimensional image. The coordinates of each component in the water–oil layered mixture can be obtained by finding the mutations of the grayscale accumulated values. 

This paper proposed the IGAVD algorithm. According to the sharp changes of the gray value at the edges of the image and gradient operators [20,45] on the basis of one-dimensional gray accumulated value data curve, the accumulated grayscale value of adjacent coordinates is processed by calculating first derivative in turn, and the absolute value of the result is obtained. Difference processing as:

(6) $$D(y)=\left|\frac{\partial C}{\partial y}\right|=\left|C(y+1)-C(y)\right|$$

where *y* = 1, 2, … *N*−1 and *N* are the number of vertical pixels in a two-dimensional plane. Because the subtraction cannot be zero, the gray accumulated value at *y* = 0 is removed and *y* = 1 is used to replace *y* = 0 as the first subtraction in our algorithm.

**Figure 5:**
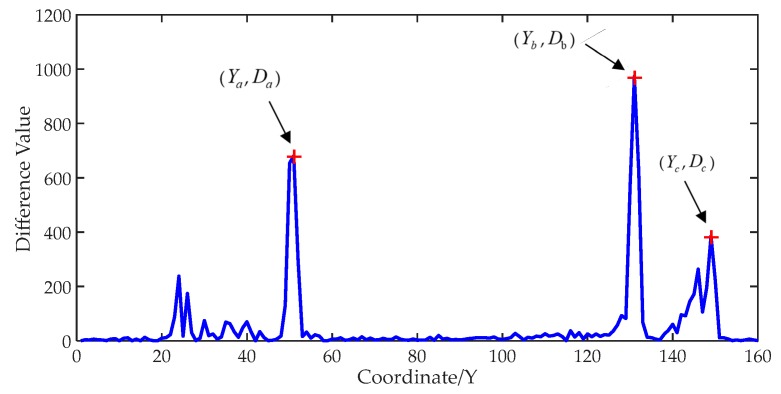
Grayscale accumulated value difference.

The results are shown in Figure 5.

After calculating the difference results, three local maxima are found by multiple traversing and polling. Operations like these can effectively prevent non-edge pixels from being detected. According to the principle of gray image edge detection, the gray difference values of edge pixels are larger than that of other regions in the image. In the entire range, the coordinates of three local maxima are the coordinates of the air–oil boundary, the oil–water boundary, and the bottom of the container. The related difference values are *D_a_*, *D_b_*, and *D_c_*. The water content and oil content in the mixture can be calculated according to the corresponding coordinates:

(7) $$Ratio_{1}=\frac{Y_{c}-Y_{b}}{Y_{c}-Y_{a}}\times 100\%,$$

(8) $$Ratio_{2}=\frac{Y_{b}-Y_{a}}{Y_{c}-Y_{a}}\times 100\%,$$

where *Ratio_1_* represents the water content in the mixture and *Ratio_2_* represents the oil content.

## 3. System Design and Implementation

In order to verify the validity of the proposed method, we further developed a miniaturized and low-cost prototype for measuring water content in crude oil.

### 3.1. Hardware Design

**Figure 6:**
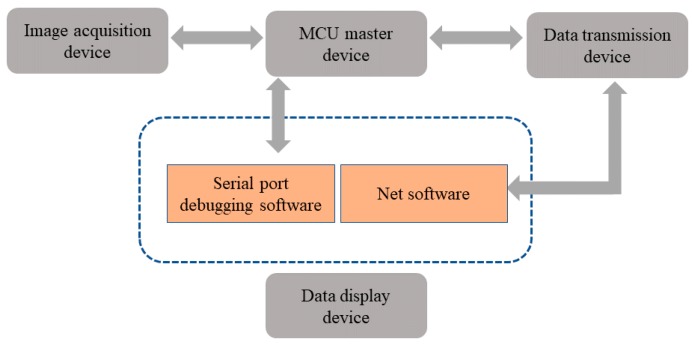
Block diagram of the overall system architecture.

The hardware system includes image acquisition device, MCU master control device, data transmission device, and data display device as shown in Figure 6.

The image acquisition device uses CMOS VGA image sensor-OV7670 produced by OmniVision (Santa Clara, CA, USA). All image processing functions of the sensor, including white balance and chroma, can be programmed through SCCB (serial camera control bus) interface, and the focal length can be adjusted manually. In the application proposed in this paper, the camera is configured to acquire images with the resolution of 320 × 240 pixels (QVGA size) in RGB565 image format. The resolution of the obtained images is consistent with the resolution of TFTLCD carried by MCU. The OV7670 image sensor is connected to the FIFO chip AL422B with memory capacity of 384 KB, and the acquired image data is stored in the AL422B chip temporarily. When using the sensor, the relevant pins are controlled according to the sequential time of the data reading by FIFO. After the IO pins of MCU read the image data in the buffer, it is further manipulated and the image is displayed on TFTLCD.

The master control device uses a 32-bit microcontroller STM32F103ZET6 chip manufactured by STMicroelectronics (Geneva, Switzerland), which is based on ARM Cortex™-M3 processor developed by ARM (Cambridge, UK). The I/O voltage is 3.3 V, and the Flash capacity is 512 K. The chip has the advantages of large capacity, low cost, high performance, and low power consumption. The control module is mainly composed of this chip, which controls image acquisition and data reading of OV7670 image sensor. The RGB565 format image is transformed into a grayscale image, and the proportion of oil in water–oil layered mixture is calculated by IGAVD algorithm. The master control device carries LED illumination compensation to assist the image data acquisition of OV7670, the on-load TFTLCD displays the collected images and adjusts the range of the images by pressing keys.

The data transmission device consists of offline data transmission and online data transmission. Offline data transmission completes the communication between MCU and PC through serial port. In this way, the results are transmitted directly to the serial port debugging software of PC without the need of LAN or WAN. The ESP8266 UART-WIFI module produced by AI-THINKER (Shenzhen, China) is used for online data transmission. The communication between MCU and PC can be accomplished by using this module through LAN or WAN, and the result calculated by MCU can be sent to the server through wireless network using TCP/IP protocol.

The data display device can display the proportional data in the PC serial port debugging software or PC monitor software made by us. Data sent directly to PC through MCU serial port is displayed in serial debugging software, and data uploaded to the server through ESP8266 UART-WIFI module is displayed on PC monitor software.

### 3.2. Software Design

**Figure 7:**
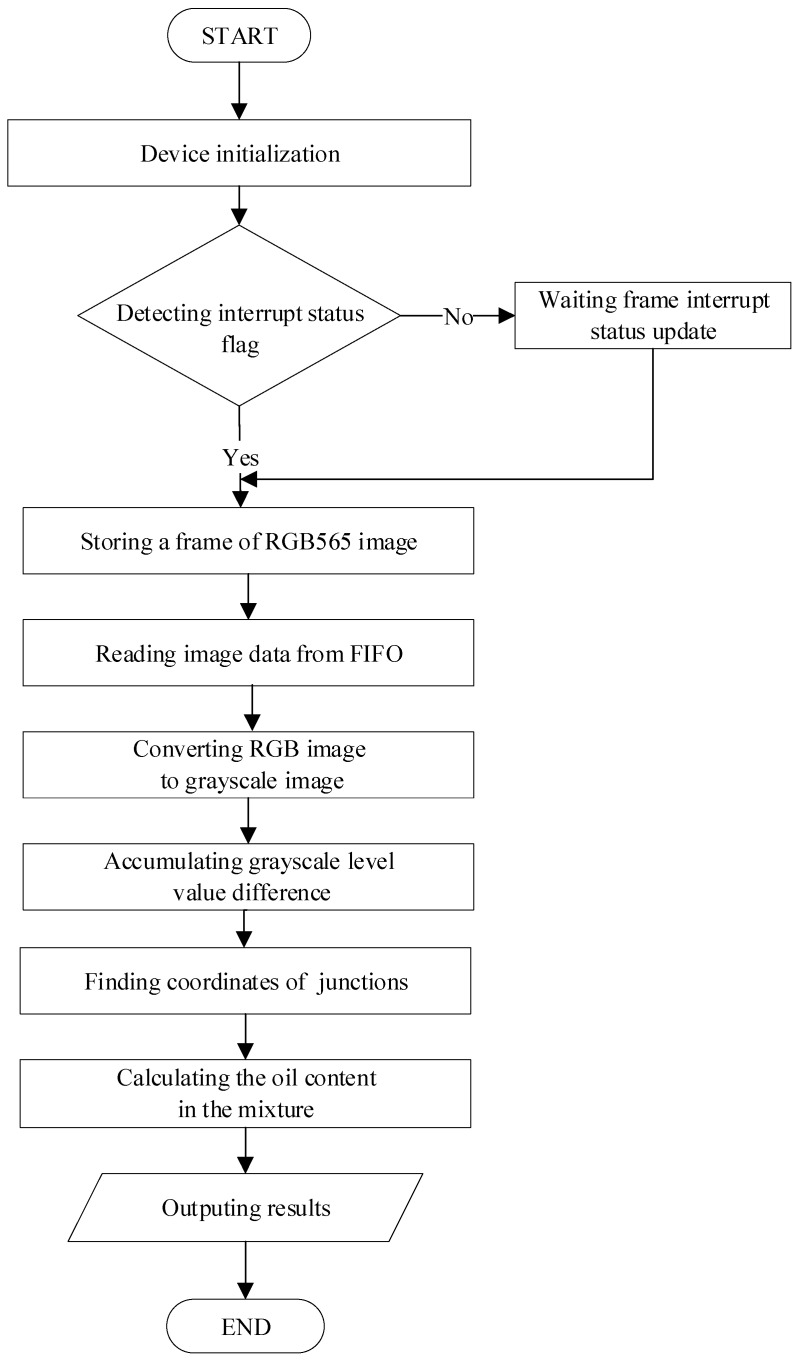
Diagram of the proposed method.

The program of IGAVD algorithm is developed on Keil uVision5 made by Keil Software (Plano, TX, USA) platform. The block diagram of the IGAVD algorithm is shown in Figure 7.

## 4. Experimental Results

Illumination is the main factor impacting the input of computer vision. In order to test the goodness and accuracy of the implemented system, we tested the best light intensity of the measuring device firstly. The experimental data in the testing procession was oil content in water–oil layered mixture.

The features such as contrast, brightness, robustness, and uniformity should be considered when choosing the light source [46]. Oil and water need to have obvious color characteristics in the measurement system, and the color characteristics of other factors in the experimental environment should be weakened substantially. As far as possible, the light source is insensitive to the position of the container, and does not produce mirror reflection on the outer wall of the container. The image contrast of the oil–water mixture collected by the sensor is blurred if the light intensity is not sufficient. In this case, the possibility of noise on the image will increase, and random light will also have an impact on the image.

In the process of validating the proposed method, it was found that the biggest factor affecting the accuracy was light brightness. To this end, the light compensation (made by LEDs, each LED’s power was 1 W) was added to the device, and the adjustable image acquisition range function was set. These configurations could avoid the noise caused by the light brightness and filter out the noise from other factors in the environment, so as to improve the measurement accuracy. The light compensation could be operated to adjust the brightness intensity and assist image sensor to complete image data acquisition when the environment lighting conditions were weak.

**Figure 8:**
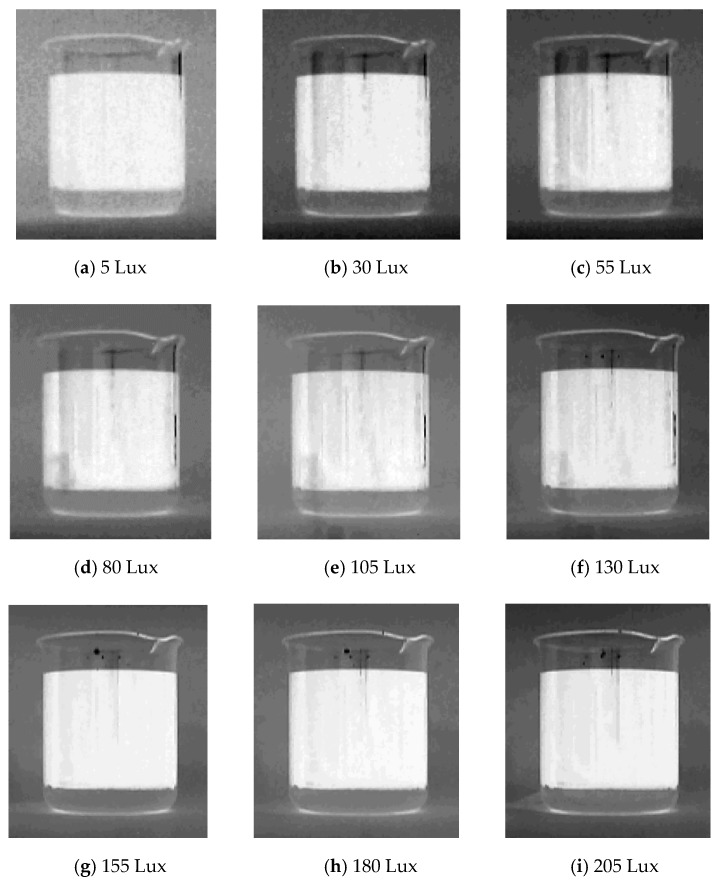
Grayscale images of the water–oil layered mixture of different illumination intensities.

Figure 8 shows the grayscale images of water–oil layered mixture collected by the image sensor under different light intensities. The lower the illumination intensities, the more noise would appear on the images.

**Figure 9:**
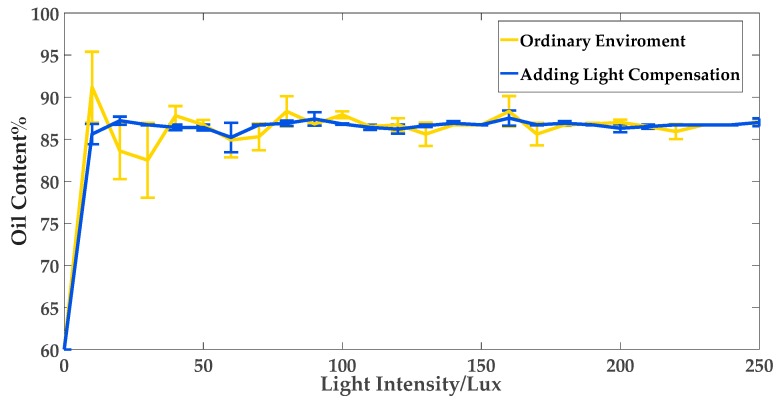
The effect of light compensation on test results.

During the experiments, the illumination of the environment was changed to verify the impact of brightness on computer vision. A total of 10 experiments were repeated under the same light intensity. The average value of ten data was taken as the final result and the standard deviation was calculated. Figure 9 and Table 1 show the test results under different illumination intensities.

Table 1 shows the comparisons whether or not to add the illumination compensation. According to Table 1, the accuracy of test results is lower when no light relatively compensation is added. The test results fluctuated less after adding illumination compensation. The experimental data shows that the light source with uniform brightness was able to bring out apparent contradistinction on the image, which met the basic light source selection requirements. During the system testing, light was provided by the experimental environment and illumination compensation, and there were no other obvious external light sources in the experimental environment.

**Figure 10:**
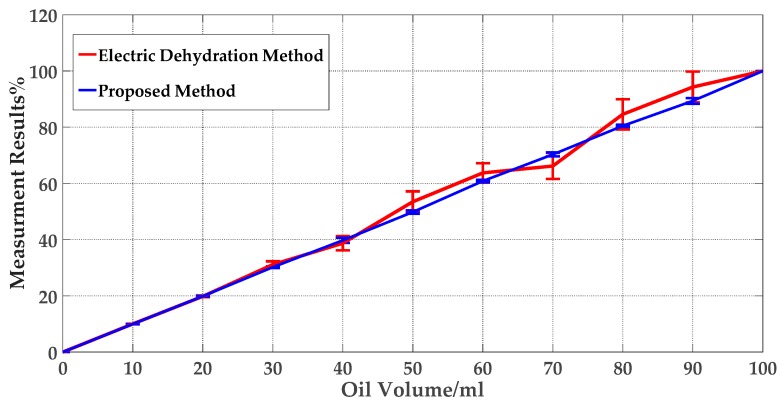
Comparisons about the proposed method and traditional method.

A total of 10 repeated tests were conducted on the sample with the same oil content under the condition of 125 Lux. Since the electric dehydration measured the water content of the crude oil by reading the volume artificially, 10 different people were asked to read the volume in the volumetric cylinder to calculate the oil content. For the proposed method, the prototype was used to conduct 10 repeated experiments on each one. As for the two different methods, we used the average values of the measuring data as the final experimental results and calculated the standard deviation as well. Figure 10 shows the comparisons for the two different methods’ test results.

In experimenting, the volume of the mixture is 100 mL and the oil volume in the sample changed from 0% to 100%. Figure 10 shows that with increasing oil content, the measurement error of the traditional method increases, and the measurement accuracy decreases gradually. Compared with the traditional method (Table 2), the proposed method is more stable, and the errors are always within 1%. Due to that the electric dehydration method detects water content by measuring the volume of water in the mixture after separation. Moreover, the measurement process of this method needs manual operation, which makes its steps complicated. To some extent, people’s subjective feelings would affect the accuracy of measurement results and lead to larger errors about the electric dehydration, but computer vision technology would not be affected by this. Therefore, the proposed method is simpler, faster, and less affected by unrelated factors such as manual operation. In addition, it has higher measurement accuracy.

Referring to previous researchers’ papers, we compared the methods proposed in this paper with distillation method, capacitance method, and shortwave absorption method. The operation process of distillation method is mainly completed by being tested in the laboratory. Artificial factors have negative impacts on the measuring results and sampling process of crude oil is random. It has good accuracy when water content is less than 1% [14]. The measurement accuracy of shortwave absorption method is about ±3% and the measurement range for water content ranges from 0% to 100% [9]. The equipment about this method has the disadvantages of complex debugging, difficult operation, and maintenance, as well as high application costs. The measurement error of capacitance method is within ±3%, and the measurement range for water content is 0%–100% [47]. This method is easy to operate and has high applicability. However, capacitance method belongs to contact measurement and sensor electrodes are vulnerable to crude oil corrosion. Therefore, this method is not suitable for a long-term measurement of water content.

**Figure 11:**
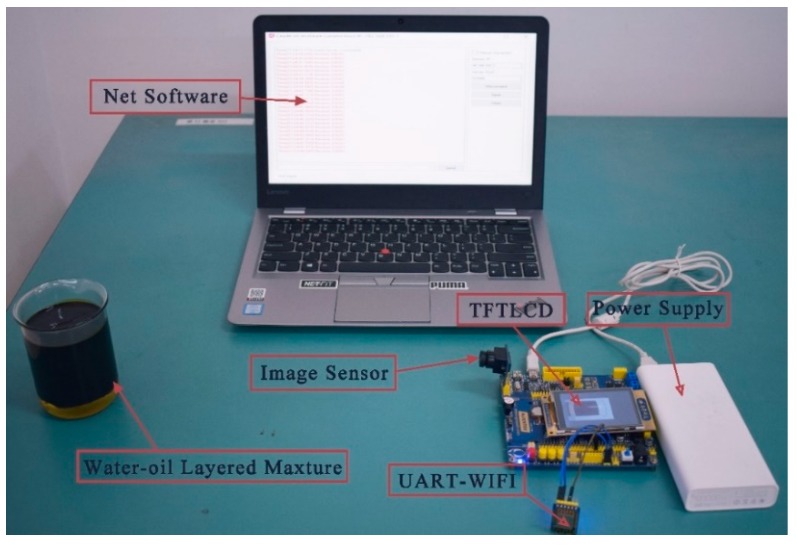
Physical photograph of the detection system.

**Figure 12:**
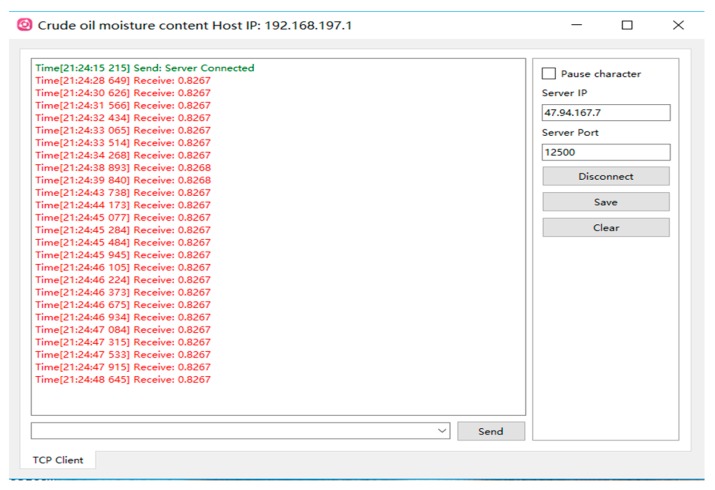
The interface of the PC monitor software.

The experimental results showed that measuring time of the prototype is no more than 3.5 s and the testing result can be updated for 1.5 times per second after the system initialization ending. The average power consumption is about 165 mW and the relative error is no more than 1.0%. Figure 11 shows the practical photograph of the measuring system. Moreover, the system can send real-time measurement results to the server through the UART-WIFI device. The interface of the host computer is shown in Figure 12. These indexes proved that the method has the advantages of wide measurement range, less susceptibility to environmental impaction, and high measurement accuracy.

## 5. Conclusions

This paper proposed a visual measurement method for water content of crude oil based on computer vision. A low-cost, universal camera was used to photograph images of water–oil layered mixture and transparent container. Then, the pixels of each component in the water–oil layered mixture were detected by IGAVD algorithm, with the water content and oil content in crude oil calculated as well. The measurement results were sent to the server in real-time to realize remote transmitting.

This method overcame the shortcomings of low automation and long measuring periods of traditional methods. It was able to realize accurate measurement for water content of crude oil, and the measuring error was less than 1%. To a certain extent, the proposed method overcame the key technical obstacles restricting the automation and informationization for measuring water content in petrochemical industry. Measuring the water content accurately is of great referential significance for determining water and oil producing horizon, predicting oil wells’ production life, and the productivity of wells. The proposed method has the characteristics of high applicability and low cost. The embedded system proposed in this paper is only a prototype of the principle. The main purpose of designing the prototype is to verify the feasibility of the proposed method and the technical indicators under different circumstances. It is not a system designed for the actual production of oil fields at present. We take into account the application of the system in actual oilfields as our future research plan. In the following research, the prototype will be improved and transformed, including optimizing the system circuit and the device’s external form, producing PCB board in order to make it more suitable for the application of the actual test scenarios.

In addition, the proposed method could be applied to the scenarios where Wang et al. [25] and Eppel et al. [26] had studied for detecting the liquid level, which is expected to provide technical reference for other measurement projects.

## Figures and Tables

**Table 1:** Measurement accuracy comparisons with or without illumination compensation.

	**Ordinary Environment**	**Add Lighting Compensation**
Light intensity when steady/Lux	95	25
Fluctuating ranges	−2.07%~+1.43%	−0.73%~+1.09%

**Table 2:** Comparison of detection results with the proposed method and traditional offline method.

	Electric Dehydration Method	Proposed Method
Measurement accuracy	−6.86% ~ +6.98%	−0.97%~+0.85%
Measurement range	0%–40%	0%–100%
Operation type	Human operation	Automatic control
